# Needs and Preferences for Peer Support and Peer Navigation Among Patients With Head and Neck Cancer: A Cross-Sectional Survey at a Canadian Cancer Centre

**DOI:** 10.1177/10732748261473344

**Published:** 2026-08-07

**Authors:** Jacqueline L. Bender, Logan Meyers, Mary Katharine Kennedy, Maria Fusaro, Jerry Lam, Lori. J. Bernstein, Amy Zhihi Liu, Melanie Woodside, Sharon Tzelnick, John Waldron, Jolie Ringash

**Affiliations:** 1 Department of Supportive Care, Princess Margaret Cancer Centre, University Health Network, Toronto, ON, Canada; 2 Dalla Lana School of Public Health, University of Toronto, Toronto, ON, Canada; 3 Department of Radiation Oncology, Princess Margaret Cancer Centre, University Health Network, Toronto, ON, Canada; 4 Department of Psychiatry, University of Toronto, Toronto, ON, Canada; 5 Department of Biostatistics, Princess Margaret Cancer Centre, University Health Network, Toronto, ON, Canada; 6 Department of Collaborative Academic Practice, Princess Margaret Cancer Centre, University Health Network, Toronto, ON, Canada; 7 Department of Otolaryngology - Head and Neck Surgery, Princess Margaret Cancer Centre, University Health Network, Toronto, ON, Canada

**Keywords:** head and neck cancer, peer support, patient navigation, peer navigation, digital health, cancer survivorship

## Abstract

**Introduction:**

People with head and neck cancers (HNC) face significant practical, functional and psychosocial barriers to supportive care, including peer support. Peer navigation may help address these challenges. This study examined the needs and preferences of patients with HNC for peer support and peer navigation.

**Methods:**

A cross-sectional survey was administered in clinic and by email to patients with HNC at a Canadian cancer centre. Descriptive statistics summarized peer support and peer navigation needs and preferences. Univariable and multivariable analyses examined factors associated with interest in receiving and providing peer navigation.

**Results:**

Participants (n=170) were on average 59 years of age (SD=13.1) and 3.2 years post-diagnosis (SD=3.4); 73.4% were male. Half of participants (n=82, 49.7%) were interested in connecting with other HNC patients at some point since diagnosis; 37.5% (n=62) had connected, and 21.5% (n=35) received peer support. Common barriers included social media privacy concerns (62%) and inconvenience of in-person programs (51%). Interest in support from a peer cancer survivor or navigator was highest before or during treatment (61%) and post-treatment (55%), and lowest in survivorship (∼20%), but increased to 47% if offered through a digital app; 43% were interested in being peer navigators. Preferred matching characteristics included cancer, treatments, specific concerns, disease stage, and age at diagnosis. Older age (p=0.02) and anxiety (p=0.02) were associated with interest in receiving peer navigation, and greater social support with being a peer navigator (p=0.02).

**Conclusion:**

Among patients with HNC surveyed, half wanted to connect with other peers, about one-third did, and only one in five received peer support. Technology-mediated peer navigation may reduce access barriers and warrants further study.

## Plain Language Summary

Many people living with head and neck cancer experience barriers to care and peer support that make it hard to get the help they need. Having someone to guide them, known as a “navigator,” could help make it easier to find care and support. In this study, researchers asked 170 patients at a Canadian cancer centre about their needs and preferences for peer support and peer navigation services. Most were about 59 years old on average, and three years post-diagnosis. About half wanted peer support, one-third connected with a peer, and only one in five actually received support The main challenges included concerns about privacy on social media and the inconvenience of attending in-person groups. Interest in support from a peer cancer survivor or peer navigator was was highest before, during, and shortly after treatment, with more than half of patients wanting this type of support. Interest was lower later in survivorship, but nearly half said they would be interested if support were offered through a digital app. Many patients (43%) were also interested in supporting others as peer navigators. Most preferred to be matched with peers who had the same type of cancer, similar treatments, or similar concerns. Older patients and those feeling anxious were more likely to want support, while people with stronger social support networks were more interested in becoming navigators for others. Overall, this study found that many patients with head and neck cancer wanted peer support, but few received it. Digital peer navigation may help improve access to support and should be studied further.

## 1. Introduction

Head and neck cancers (HNC) (e.g., cancers of the oral/nasal cavity, pharynx, larynx) account for 5% of all cancer cases globally,^1^ and 8,100 new cases, and 2,100 deaths in Canada each year.^2^ The incidence and mortality of HNC varies by geographic region, socioeconomic characteristics, and lifestyle factors. HNC is more common in men than women with an incidence ratio of approximately 3:1, adults over 50 years of age, people with lower socioeconomic status and people of East and South Asian descent.^3-8^ While tobacco and alcohol use remain significant risk factors, there is an increasing incidence of human papilloma virus (HPV)-related HNC, especially among younger populations.^3^

HNCs and the treatments currently required to control them result in significant practical and functional impairments.^9^ Common impairments include difficulties in mastication, swallowing, and speaking; loss of smell, taste, and hearing; sexual health and body image challenges; memory and attention difficulties; and physical disfigurement, pain, and fatigue.^4-7,10,11^ These challenges reduce participation in social and occupational activities, resulting in social isolation and psychosocial distress, and contribute to worse health-related quality of life in HNC survivors relative to other cancer populations.^6,12^ Many HNC patients lack adequate support^9^ and experience barriers to care (e.g. stigma, lower socioeconomic status and complex survivorship care involving multiple providers),^13-15^ leading 60-70% to report unmet supportive care needs after treatment.^8^ Peer support and patient navigation were identified as priorities at an institutional retreat attended by HNC patients, caregivers and healthcare professionals that may address existing gaps in supportive care.

Peer support - social support (e.g. informational, emotional, practical) from a person with relevant lived experience^16^ - may be particularly important for patients with HNC who are at higher risk of isolation given the impact of the disease on social functioning.^12^ Despite this, there has been limited prior research on the peer support needs or experiences of patients with HNC. A 2020 systematic review identified ten studies of peer support in HNC, the most common form of which was in-person support groups.^17^ In-person and online support groups can help patients with HNC discuss their feelings and the challenges they face, leading to a reduced sense of isolation and distress and improved quality of life.^18-20^ However, group-based peer support may not meet everyone’s needs. Groups may not contain the right composition of diagnoses, treatments or lived experiences, and they may not offer the personalized support needed to overcome barriers to care, particularly for structurally marginalized patients.^21-23^

Patient navigation is an individualized intervention led by clinical, lay or peer (cancer survivor) navigators that aims to overcome barriers to care, facilitate timely access to healthcare services, and reduce healthcare disparities.^24^ The most recent systematic review of cancer patient navigation found strong evidence for improved screening rates, timeliness of diagnostic resolution and treatment initiation, and emerging evidence for improved patient satisfaction and quality of life post-treatment.^24^ However, only two randomized controlled trials examined patient navigation specifically for patients with HNC. Both were led by nurse navigators and demonstrated improvements in timeliness of adjuvant radiation therapy treatment,^25^ and quality of life and symptom management.^26^ No studies examined peer navigation in HNC and only three studies evaluated technology-enabled navigation solutions.^24^

Peer navigation integrates peer support and patient navigation by engaging trained cancer survivors as navigators. Unlike traditional peer support, peer navigation provides structured, goal-oriented support aimed at reducing barriers to care, promoting timely access to services, and addressing patients supportive care needs. Technology has the potential to extend the reach of peer navigation by overcoming access barriers and supporting navigators with administrative tasks.^27^ We previously developed a digital peer navigation intervention that was shown to improve patient activation and quality of life and reduce unmet supportive care needs in patients treated for prostate cancer.^28,29^ An adapted version of this digital peer navigation program, tailored for patients with HNC, may help address their peer support and navigation needs. Before adapting the intervention for the HNC population, a needs assessment is required. Therefore, this study aimed to examine the peer support and peer navigation needs and preferences of patients with HNC.

## 2. Methods

### 2.1. Study Design

A cross-sectional survey design was employed and reported in accordance with the Strengthening the Reporting of Observational Studies in Epidemiology (STROBE) reporting guidelines.^30^ Ethical approval was obtained from the University Health Network (UHN) Research Ethics Board (CAPCR ID: 19-5361). The study was conducted in accordance with the Helsinki Declaration of 1975, as revised in 2024. All participants provided informed consent.

### 2.2. Participants

#### 2.2.1. Setting and Population

The survey was distributed to eligible patients at the Princess Margaret Cancer Centre in the Wharton Head & Neck Clinic in Toronto, Ontario, Canada between June 1 and August 31, 2019. This HNC clinic treats approximately 800 unique patients per year with HNC affecting the lips and mouth, oropharynx, hypopharynx, nasopharynx, larynx, nasal/sinus, and salivary glands, as well as complex skin cancers of the head and neck; surgical treatment is provided to patients with cancers of the thyroid. At the time the study was conducted, there were no peer support or navigation services specifically for patients with HNC at the institution. A nurse-led online peer support group was launched for HNC patients in 2023.

#### 2.2.2. Eligibility Criteria

Individuals were eligible to take part in this study if they had a confirmed diagnosis of HNC, were 18 years of age or older, could read and speak English, and were able to provide informed consent. Patients at any stage of treatment or survivorship were eligible. We excluded patients if they only had thyroid cancer, or skin cancers of the head and neck, which are not considered head and neck cancers.

### 2.3. Data Collection

#### 2.3.1. Recruitment Procedures and Sample Size

Eligible patients were asked to complete a 25-to-30-minute questionnaire on paper or online. Study information was shared with clinicians and clinic administrators, study posters and post-cards were placed in common areas, and clinicians shared study postcards with patients. Research assistants consecutively approached patients in-clinic after patients expressed interest in participating to clinic staff. In addition, study information was sent via email to the HNC Survivorship Program email list which was comprised of patients who had previously agreed to be contacted about HNC Survivorship program activities and research opportunities. We aimed to recruit a sample of at least 150 participants to support a robust multivariable analysis with 10 independent variables (e.g. min of 10 participants per variable).^31^

#### 2.3.2. Questionnaire

The questionnaire was based on a previous questionnaire that we developed to examine the peer support and peer navigation needs and preferences of adolescents and young adults with cancer (AYAs).^32^ Adaptations included modifying clinical questions to capture HNC diagnoses and treatments and replacing AYA specific measures with HNC-specific measures. This study reports on data collected from the following sections (A) background (e.g., age, education, income): and disease characteristics (e.g., diagnosis, date of diagnosis, treatments received) consisting of 25 questions; (B) needs, experiences and preferences for peer support and interest in and preferences for peer navigation consisting of 20 questions; and (C) psychosocial wellbeing, including anxiety [7-item Generalized Anxiety Disorder (GAD-7)]^33^, social support [10-item Social Provisions Scale (SPS-10)], 3^34^ and loneliness (20-item UCLA Loneliness Scale).^35^ Data on diagnosis and treatment was collected from the prospectively populated Anthology of Outcomes database,^36^ supplemented by medical records for patients recruited in-clinic.

### 2.4. Data Analysis

Statistical analysis was performed using SPSS version 28. Means and standard deviations were reported for continuous variables and frequency, and percentages were reported for categorical variables. Logistic regression analyses were conducted to identify factors associated with two binary outcomes: (i) interest in being connected with a peer navigator (ii) interest in being a peer navigator. Variables identified a priori (age, gender, education, relationship status, race/ethnicity, time since diagnosis, HPV status, cancer treatment, anxiety, and social support) were included in multivariable models. Multicollinearity was assessed using variance inflation factors (VIF < 5). Odds ratios (OR) with 95% confidence intervals (CI) were reported. Missing data were handled by listwise deletion. In addition, a subgroup analysis was performed using logistic regression to explore differences in the prioritization of peer matching characteristics by race and the importance of peer navigator training based on interest in being a peer navigator. Responses regarding importance were dichotomized as not important vs. important (somewhat important, very important, not sure). P-values < 0.05 were considered statistically significant.

## 3. Results

### 3.1. Participant Characteristics

**Figure 1.**
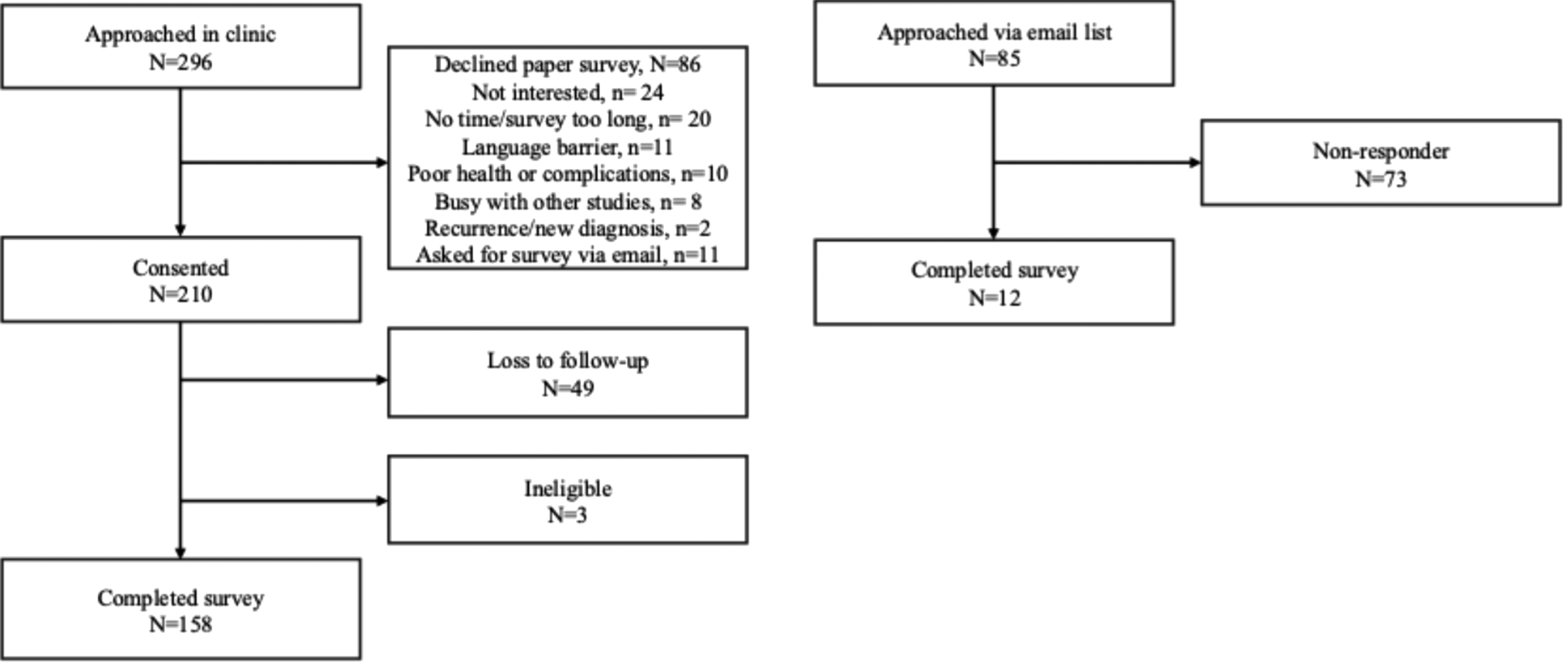
Recruitment flow diagram

One hundred and seventy patients with HNC completed the questionnaire (Figure 1). Participants were a mean of 59.4 years of age (SD 13.1) and 3.2 years (SD 3.4) post-diagnosis. Most were diagnosed with cancer of the oropharynx (n= 69, 40.6%). About three-quarters (n=125, 74.9%) identified as male and white (n=129, 76.8%). Sixty-two percent were born in Canada (n=105) and 72% (n= 121) had completed college or university (Table 1).

**Table 1.** Participant Characteristics

Characteristic (*n*)	Category	Count (%), unless otherwise specified
Age (years), mean (SD) (*n* = 170)	​	59.4 (13.1)
Time since diagnosis (years), mean (SD), (*n* = 168)	​	3.2 (3.4)
Cancer diagnosis (*n* = 170)	Oropharynx	69 (40.6)
	Oral cavity	28 (16.5)
	Nasopharynx	17 (10)
	Salivary glands	16 (9.4)
	Larynx	12 (7.1)
	Skin of face/scalp	9 (5.3)
	Nasal cavity and paranasal sinus	6 (3.5)
	Cancer of unknown primary	4 (2.4)
	Sarcoma including dermatofibrosarcoma protuberans	4 (2.4)
	Eye (including lacrimal gland and orbit)	3 (1.8)
	Esophagus	3 (1.8)
	Paraganglioma	1 (0.6)
	Hypopharynx	1 (0.6)
Cancer treatments undergone or currently receiving (*n* = 168)	Radiation therapy	107 (63.7)
	Surgery	66 (39.3)
	Chemotherapy	6 (3.6)
	Other	1 (0.6)
	None	1 (0.6)
Sex (*n* = 169)	Male	124 (73.4)
	Female	45 (26.6)
Gender (*n* = 167)	Man	125 (74.9)
	Woman	42 (25.2)
Race/Ethnicity (*n* = 168)	White	129 (76.8)
	East Asian	18 (10.7)
	South Asian	10 (6)
	Black/African Canadian	3 (1.8)
	Middle Eastern	3 (1.8)
	Filipino	3 (1.8)
	Latino/Hispanic	1 (0.6)
	Mixed race	1 (0.6)
Country of birth (*n* = 169)	Canada	105 (62.1)
	Other	64 (37.9)
Spoken language at home (n=151)	English	133 (88.1)
	Other	18 (11.9)
Education completed (*n* = 168)	College/university	121 (72)
	High school and/or less	47 (28)
Relationship status (n=168)	With a partner (e.g. married, common law)	132 (78.6)
	Without a partner (e.g., single, divorced, widowed)	36 (21.4)
Living setting (*n* = 166)	Urban or suburban (city)	132 (79.5)
	Town or rural (country)	34 (20.5)
Employment status (*n* = 169)	Retired	66 (39.1)
	Working full time	58 (34.3)
	Working part time	25 (14.8)
	Unemployed	11 (6.5)
	Long-term disability	8 (4.7)
	Did not specify	1 (0.6)
Household income (*n* = 158)	More than $80,000	68 (43)
	$40,001-$80,000	43 (27.2)
	$20,000-$40,000	28 (17.7)
	Less than $20,000	19 (12)

### 3.2. Peer Support Needs and Preferences

Approximately fifty percent of survey respondents (n=82, 49.7%) reported that they wanted to connect (e.g. meet in person, talk on the phone, or online) with other patients with HNC at some point since their diagnosis (Table 2). Two-thirds (n=109, 65.7%) had not talked to a healthcare professional about peer support. Of those who had, most (n=45, 27.1%) relied on their healthcare professional to initiate the conversation. Only 10.7% (n=18) received a referral to a peer support program.

**Table 2.** Peer Support Needs and Preferences

Variable (*n*)	Category	Count (%)
Since your diagnosis, have you ever wanted to connect (meet in person, talk on the phone or online) with other patients with HNC? (*n* = 165)	Yes	82 (49.7)
	No	83 (50.3)
Have you ever talked to a healthcare professional about peer support? (*n* = 166)	No	109 (65.7)
	Yes, a medical professional initiated the conversation	45 (27.1)
	Yes, I initiated the conversation	12 (7.2)
Have you ever received a referral to a peer support program? (*n* = 168)	Yes	18 (10.7)
	No	150 (89.3)
Have you ever connected with other patients with HNC? (n=165)	Yes	62 (37.5)
	No	103 (62.4)
Have you connected with other patients with HNC using any of the following methods? (*n* = 50)	Friends and relatives	20 (40)
	Social media/Online discussion forum	13 (26)
	Health-care professional	12 (24)
	In-person support group	10 (20)
	Hospital Waiting room or Lodge	9 (18)
	Cancer organization	6 (12)
	Conference, Workshop or Committee	6 (12)
	Digital apps that connect HNC patients	2 (4)
	Other- Church groups	1 (2)
	Other- Patients of mine	1 (2)
Can you count on an HNC patient to…		
Provide you with good information or advice regarding a problem? (n=157)	I would like/or I would like more of this type of support	48 (30.6)
	I currently have this type of support	13 (8.3)
	Not needed	96 (61.1)
Provide you with emotional support? (n=157)	I would like/or I would like more of this type of support	46 (29.3)
	I currently have this type of support	10 (6.4)
	Not needed	101 (64.3)
Provide you with practical help with things, such as daily chores, childcare or getting to appointments? (n=156)	I would like/or I would like more of this type of support	18 (11.5)
	I currently have this type of support	3 (1.9)
	Not needed	135 (86.5)
Hang out with and perform normal social things? (n=155)	I would like/or I would like more of this type of support	22 (14.2)
	I currently have this type of support	6 (3.9)
	Not needed	127 (81.9)
How satisfied are you with the peer support received from other HNC patients? (n=163)	Very dissatisfied	2 (1.2)
	Dissatisfied	2 (1.2)
	Neutral	15 (9.2)
	Satisfied	11 (6.7)
	Very satisfied	5 (3.1)
	NA, I did not receive support from another HNC patient	128 (78.5)
Would you be interested in being connected with a peer cancer survivor (at the time the survey was conducted) (n=163)	Yes	36 (22.1)
	No	127 (77.9)
If no: Why not? (n=125)	I don’t think I need support	61 (48.8)
	I believe I have adequate support	55 (44.8)
	I don’t like to talk about my problems	14 (11.2)
	Other- completed treatment	1 (0.008)
	Other- communication challenges	1 (0.008)
	Other- don’t speak English well	1 (0.008)
	Other- no time	3 (0.024)

*Note*. A peer cancer survivor was defined as a patient with HNC who has been through the cancer experience, also known as a peer mentor.

Approximately one-third of participants (n=62, 37.5%) reported connecting with other HNC patients at some point since their diagnosis. Among those who indicated how they connected with other HNC patients, the most common route was through friends and family (n=20; 40%).

When asked about satisfaction with peer support, most individuals (n=128, 78.5%) reported that they had not received peer support from another patient with HNC. Among the 35 individuals (21.5%) who had received peer support from another patient with HNC, fewer than half (n=16, 45.7%) were satisfied or very satisfied with the support received.

Participants indicated a greater need for informational (n=48; 30.6%) and emotional support (n=46, 29.3%) from other HNC patients, than companionship (n=22, 14.2%) or practical (n=18, 11.5%) support.

At the time the survey was conducted, which was when most were on average 3 years post-diagnosis, 22% (n=36) expressed interest in being connected with a peer cancer survivor. Among those not interested, most either felt that they did not need support (n=61, 48.8%) or that they had adequate support (n=55, 44.8%).

### 3.3. Barriers to Peer Support

The most common barriers to peer support (≥ ∼50% endorsing as a “problem”) were online privacy concerns and logistical access barriers: 62% (n=85) had privacy concerns related to discussing health on social media and 51% (n=69) found in-person programs inconvenient to attend (Table 3). Approximately 40% had interpersonal concerns about participating in peer support, including concerns about getting close to someone who might die (n=62, 46%), being around negative minded cancer patients (n=59, 44%), discomfort attending in-person support groups (n=58, 42%), and difficulty finding another patient with HNC with whom they could relate (n=57, 42.5%). The least commonly reported barriers to peer support (≤ ∼20% endorsing as a “problem”) were technology and preference-related: 18% (n=25) reported difficulty using a computer or mobile device, 18% (n=24) preferred to connect with health peers rather than cancer patients, and 12.5% (n=17) did not have access to a computer or mobile device.

**Table 3.** Barriers to Peer Support

Variable (*n*)	Count (%)		
	Problem	No problem	Not sure
Privacy concerns related to discussing health on social media (*n* = 137)	85 (62)	42 (30.7)	10 (7.3)
In-person programs were not convenient to attend (*n* = 135)	69 (51.1)	39 (28.9)	27 (20)
Concern about getting close to someone who might die (*n* = 136)	62 (45.6)	59 (43.3)	15 (11)
Concern about being around negative minded cancer patients (*n* = 134)	59 (44)	60 (44.8)	15 (11.2)
Discomfort attending in-person support programs (*n* = 138)	58 (42)	60 (43.5)	20 (14.5)
Difficulty finding another HNC patient to relate to (*n* = 134)	57 (42.5)	36 (26.9)	41 (30.6)
Uncertainty about where or how to find other HNC patients (*n* = 138)	55 (39.9)	42 (30.4)	41 (29.7)
HNC-specific in-person programs were hard to find (*n* = 134)	48 (35.8)	31 (23.1)	55 (41)
Concern about hearing emotionally difficult stories (*n* = 136)	48 (35.3)	75 (55.1)	13 (9.6)
Difficulty using social media (*n* = 138)	45 (32.6)	84 (60.9)	9 (6.5)
Difficulty using a computer or mobile device (*n* = 138)	25 (18.1)	106 (76.8)	7 (5.1)
Preference to connect with healthy peers rather than cancer patients (*n* = 133)	24 (18)	83 (62.4)	26 (19.5)
Lack of access to computer or mobile device (*n* = 136)	17 (12.5)	112 (82.4)	7 (5.1)

*Note*. Barriers to peer support were categoized as no problem, a problem (somewhat of a problem, big problem) or not sure.

### 3.4. Peer Navigation Needs and Preferences

At the time the survey was conducted (which was when most were on average 3 years post-diagnosis), 19% (n=30) expressed interest in being connected with a peer navigator, defined as a cancer survivor trained to help others through the cancer experience (Table 4). Among those not interested, most either felt that they did not need support (n=61, 50.8%) or that they had adequate support (n=53, 44.2%).

**Table 4.** Peer Navigation Needs and Preferences

Variable (*n*)	Category	Count (%)
**Interest in Receiving or Offering Peer Navigation**		
Would you be interested in being connected with a peer navigator (at the time the survey was conducted) (*n* = 158)	Yes	30 (19)
	No	128 (81)
If no: Why not? (*n* = 120)	I don’t think I need support	61 (50.8)
	I believe I have adequate support	53 (44.2)
	I don’t like to talk about my problems	13 (10.8)
	My English isn’t good enough	2 (1.6)
	Difficult to travel	1 (0.8)
	I don’t need support, but I can give support	1 (0.8)
	Not sure	1 (0.8)
Would you be interested in being a peer navigator (*n* = 151)	Yes	65 (43.1)
	No	86 (57)
Would you be willing to attend peer navigator training to become a peer navigator? (*n* = 147)	Yes	60(40.8)
	No	87(59.2)
How important is it for you to be connected with a peer navigator who has received training to support others through the cancer experience? (*n* = 163)	Not important	71 (43.6)
	Slightly to very important	60 (36.8)
	Unsure	32 (19.6)
**Timing, Support, and Communication Preferences**		
When would it be most helpful to be connected with a peer cancer survivor/navigator? (n=136)	During treatment	83 (61)
	Before treatment	82 (60.3)
	After treatment	76 (55.9)
	If cancer recurs or spreads	65 (47.8)
	During diagnosis	59 (43.4)
	No interest	6 (3.6)
	Not sure/Did not specify	4 (3)
	While the disease is stable	1 (0.7)
	Don’t want a digital connection	1 (0.7)
What type of support would you like to receive from a peer cancer survivor/navigator?	Informational support (n=145)	115 (79.3)
	Emotional support (n=143)	109 (76.2)
	Practical support (n=140)	79 (53.6)
	Social companionship support (n=142)	72 (50.7)
How would you like to communicate with a peer cancer survivor/navigator? (n=133)	Email (n=133)	63 (47.4)
	In-person (n=133)	62 (46.6)
	Telephone (n=133)	52 (30.6)
	Social media (n=133)	19 (14.3)
	Video (n=133)	15 11.3)
**Digital Peer Navigation**		
Would you be interested in using a **digital app** to connect and communicate with a peer cancer survivor/navigator (at the time the survey was conducted)? (*n* = 149)	Yes	70 (47)
	No	79 (53)
If no: Why not? (*n* = 79)	Did not specify	35 (44.3)
	Not interested	21 (26.6)
	Not good with technology/No cell phone	15 (19)
	Prefer in-person either initially or entirely	3 (3.8)
	No time	2 (2.5)
	Prefer email	1 (1.3)
	Privacy concerns	1 (1.3)
	Don’t like to talk about my problems	1 (1.3)
In the digital app, how would you want to communicate with a peer cancer survivor/navigator through a digital app (n=105)	One-on-one	55 (52.4)
	In a group with other HNC patients,	55 (52.4)

*Note*. A peer navigator was defined as a cancer survivor who has been trained to help others through the cancer experience. Participants were informed that they could use the digital app to view the profiles of HNC patients that match their selection criteria (e.g. age, cancer, diagnosis) and choose the person they would like to talk to. Participants were also informed that they would be provided training and professional support to be a peer navigator. They were also informed that the role of a peer navigator was to: assist patients in identifying needs and overcoming barriers to getting their needs met; discuss experiences and validate feelings and concerns and empower patients by working with them to identify their own strengths, abilities and coping strategies. Type of support wanted from a peer navigator was dichotomized as not at all important vs important (slightly important, moderately important, important, very important).

Forty-three percent of participants (n=65) were interested in being a peer navigator and 41% (n= 60) were willing to attend a training program to become a peer navigator. However, 44% (n= 71) rated training for the peer navigator role as not important. Participants who wanted to be peer navigators were 4.2 times more likely to rate training important (OR=4.2, 95%CI:2.06, 8.57), p<0.001).

### 3.5. Timing, Support and Communication Preferences

Interest in support from a peer cancer survivor/navigator was highest during or before treatment (n=83 or 82, 61%) and post-treatment (n=76, 55%), compared with during recurrence/ progression (n= 65, 47%) or during diagnosis (n=59, 43%).

More participants wanted informational support (n=115, 79.3%) and emotional support (n=109, 76.2%) from a peer cancer survivor/navigator, than practical or companionship support (56.4% and 50.7% respectively).

Most participants would prefer to communicate with a peer cancer survivor/navigator by email (n=63, 47.4%) or in-person (n=62, 46.6%), rather than by telephone (n=52, 30.6%). Participants were least interested in communicating with a peer cancer survivor/navigator on social media (n=19, 14.3%) or by video (n=15, 11.3%).

Nearly half (n=70, 47%) were interested in using a digital app to connect and communicate with a peer cancer survivor/navigator, where they could view the profiles of people that matched their criteria, and choose the best fit. Among those not interested, most did not specify the reason (n=35, 44.3%), or said they were not interested (n=21, 26.6%); 19% (n=15) indicated they were not comfortable with technology or did not own a cellphone.

An equal proportion indicated that they would like to communicate with a peer cancer survivor/navigator one-on-one or in a group with other HNC patients in a digital app (52.4%).

### 3.6. Preferred Peer Matching Characteristics

The most important peer matching characteristics (≥ ∼50% endorsing as “important”) were related to clinical and situation specific characteristics, such as: cancer type (75.5%), treatments received (69.8%), specific concerns (68.6%), stage of disease (56.3%) and age at diagnosis (51.4%) (Table 5). The least important peer matching characteristics (≤ ∼20% endorsing as a “important”) were education (n=24, 17.5%), race/ethnicity (n=22, 16.1%), and relationship status (n= 18, 13%). However, participants who identified as racialized were 2.5 times more likely to rate matching based on race as important than those who identified as white (OR=2.57, 95% CI: 1.03-6.45, p = 0.04).

**Table 5.** Peer Matching Characteristics

Variable (n)	Count (%)		
	Important	Not important	Not sure
Type of cancer (n = 139)	105 (75.5)	27 (19.4)	7 (5)
Treatment received (n = 139)	97 (69.8)	35 (25.2)	7 (5)
Specific concerns (n = 137)	94 (68.6)	35 (25.5)	8 (5.8)
Stage of disease (n = 135)	76 (56.3)	46 (34.1)	13 (9.6)
Age at diagnosis (n = 142)	73 (51.4)	64 (45.1)	5 (3.5)
Similar geographic region (n = 137)	67 (48.9)	61 (44.5)	9 (6.6)
Treating hospital (n = 140)	68 (48.6)	62 (44.3)	10 (7.1)
Current age (n = 137)	64 (46.7)	69 (50.4)	4 (2.9)
Coping style (n = 135)	55 (40.7)	71 (52.6)	9 (6.7)
Personality style (n = 137)	54 (39.4)	75 (54.7)	8 (5.8)
Gender (n = 138)	43 (31.2)	88 (63.8)	7 (5.1)
Religion/spirituality (n = 135)	37 (27.4)	92 (68.1)	6 (4.4)
Hobbies/interests (n = 137)	36 (26.3)	94 (68.6)	7 (5.1)
Sexual orientation (n = 137)	30 (21.9)	99 (72.3)	8 (5.8)
Education (n = 137)	24 (17.5)	108 (78.8)	5 (3.6)
Race/ethnicity (n = 137)	22 (16.1)	108 (78.8)	7 (5.1)
Relationship status (n = 137)	18 (13.1)	113 (82.5)	6 (4.3)

*Note*. Peer matching characteristics were categorized as not important, important (somewhat important, very important) or not sure.

### 3.5. Factors Associated With Interest in a Peer Navigator and Being a Peer Navigator

Factors associated with interest in being connected with a peer navigator were older age (OR 1.03, 95%CI: 1.00,1.07, p=0.05) and higher anxiety (OR 1.15, 95% CI: 1.06,1.26, p=0.002) (Table 6). In the multivariable analysis, older age (adjusted OR=1.05, 95% CI: 1.01-1.10, p=0.02) and higher anxiety (adjusted OR=1.15, 95% CI: 1.02-1.29, p=0.002) remained significant predictors. Factors associated with interest in being a peer navigator were higher education (OR 1.49, 95%CI:1.14,5.30, p=0.02) and greater social support (OR 1.07, 95% CI: 1.00,1.14, p=0.049). In the multivariable analysis, social support (OR=1.12, 95% CI: 1.02-1.22, p=0.02) remained a significant predictor.

**Table 6.** Factors Associated With Interest in a Peer Navigator and Being a Peer Navigator

Variable (reference category)	Interest in a peer navigator					Interest in being a peer navigator				
	Unadjusted			Adjusted (N = 115)		Unadjusted			Adjusted (N = 113)	
	N	OR (CI)	p-value	OR (CI)	p-value	N	OR (CI)	p-value	OR (CI)	p-value
Age	158	1.03 (1.00,1.07)	**0.05**	1.05 (1.01,1.10)	**0.02**	151	1.00 (0.98,1.03)	0.97	1.00 (0.97,1.04)	0.82
Gender (Man) Woman	155	1.49 (0.61,3.60)	0.38	1.47 (0.43,5.01)	0.54	148	1.49 (0.70,3.18)	0.31	1.93 (0.68,5.46)	0.21
Education (High school or less) College/University	157	1.25 (0.49,3.17)	0.64	1.55 (0.47,5.08)	0.47	149	2.45 (1.14,5.30)	**0.02**	1.96 (0.79,4.87)	0.15
Relationship Status (No partner) Has a partner	158	1.18 (0.44,3.17)	0.74	1.44 (0.40,5.19)	0.58	151	2.20 (0.97,5.01)	0.06	1.63 (0.58,4.54)	0.36
Race (White) Racialized	157	0.85 (0.32,2.29)	0.75	0.83 (0.25,2.84)	0.77	150	0.58 (0.26,1.31)	0.19	0.86 (0.30,2.49)	0.78
Time since diagnosis	156	0.99 (0.27,3.54)	0.98	1.06 (0.69,1.63)	0.79	147	1.31 (0.42,4.13)	0.64	1.08 (0.76,1.54)	0.67
HPV status (No) Yes	134	1.02 (0.44,2.37)	0.96	1.09 (0.37,3.18)	0.88	129	1.84 (0.91,3.70)	0.09	2.18 (0.90,5.25)	0.08
Treatment										
Radiation (No) Yes	156	0.58 (0.26,1.34)	0.20	​	​	149	0.71 (0.37,1.39)	0.32	​	​
Surgery (No) Yes	156	1.49 (0.66,3.41)	0.34	​	​	149	1.21 (0.62,2.34)	0.58	​	​
Anxiety	146	1.15 (1.06,1.26)	**0.002**	1.15 (1.02,1.29)	**0.02**	144	1.03 (0.95,1.11)	0.47	1.07 (0.95,1.19)	0.26
Social Support	143	0.99 (0.93,1.06)	0.8	1.04 (0.94,1.15)	0.49	140	1.07 (1.00,1.14)	**0.049**	1.12 (1.02,1.22)	**0.02**

*Note.* HPV is human papilloma virus.

## 4. Discussion

### 4.1. Summary

This study investigated the peer support and peer navigation needs and preferences of patients with HNC at a large urban cancer centre in Canada who were on average 3 years post-diagnosis. Half of participants (49.7%) reported that they wanted to connect with other HNC patients at some point since diagnosis; 37.5% connected with other HNC patients and 21.5% received peer support. Interest in support from a peer survivor/navigator was highest before or during treatment (61%) and post-treatment (54%), and lowest in survivorship (∼20%), but increased to 47% if offered through a digital app; 43% were interested in being peer navigators. Patients who were older or more anxious were more likely to be interested in receiving support from a peer navigator, while those who had greater social support were more likely to be interested being peer navigators. These findings show a need for peer support among a considerable proportion of this population and practical barriers to accessing such support. They also suggest an interest in peer support and peer navigation that is supported by technology and factors that may predict interest in receiving and providing peer navigation.

### 4.2. Perceived Need and Awareness of Peer Support

Although 49.7% of the sample reported that they wanted to connect with other patients with HNC at some point since diagnosis, only 34% discussed their peer support needs with a healthcare professional, and 27% relied on their healthcare professional to initiate the conversation. Given that only 11% of patients surveyed reported receiving a referral to a peer support program and most patients connected with other patients with HNC through family and friends, these findings may reflect limited availability or awareness of peer support options. At the time of the survey, there were no peer support programs specifically for patients with HNC at the hospital Since then, a nurse-led online peer support group was launched for HNC patients. Collectively, these findings suggest that routine screening for peer support needs, coupled with mechanisms to connect patients to available peer support services may be benefit this population.

### 4.3. Barriers to Accessing Peer Support

Despite 49.7% wanting to connect with other HNC patients since diagnosis, only 37.5% had connected with another HNC patient and 21.5% had received peer support. The perceived need for peer support in this sample was higher than in a survey of 26 HNC survivors which was reported to be 27%, but access was similar at 20%.^37^ The most common barriers to peer support reported by participants in this study were social media privacy concerns and inconvenience of in-person programs, endorsed by over 50% of the sample. Approximately 40%, also endorsed interpersonal concerns such as fear of getting close to someone who might die, negative minded patients, and difficulty finding another patient with whom they could relate. Whereas the least common barriers, endorsed by less than 18%, were mainly technology-related, including lack of access to or difficulty using a computer. In comparison, in a focus group study of 40 Danish patients with HNC, common reasons for not communicating with peers during treatment was lack of energy, uninviting environment, and fear of facing a peer with more progressive disease or symptoms.^38^ These barriers may be amplified for patients with cancer-related cognitive, psychosocial, or fatigue symptoms, where peer support could provide practical strategies for managing memory or attention deficits which may impact how they manage their survivorship care.

### 4.4. Timing of Need for Peer Support and Peer Navigation and the Role of Technology

Need for peer support and peer navigation varied over the course of the cancer care continuum. Interest in support from a peer cancer survivor/or navigator was highest during or before treatment (∼61%) and post-treatment (55%). Interest was lower during recurrence or progression (47%) and at during diagnosis (43%), and lowest at the time of the survey which was when most were later in the survivorship trajectory (∼20%). Two other studies that examined timing of participation in peer support among patients with HNC, also found that participation declined in the survivorship phase. Oskam found that 16% of 26 patients with HNC participated in peer support (individual or group-based) during treatment, dropping to 4% 8 to 11 years after treatment.^37^ While Caroll-Alfano found that 78% of 200 patients with HNC participated in support groups (in-person or online) within 3 years post-treatment, dropping to 63% 10 or more years after treatment.^39^

However, interest in peer support or peer navigation during the survivorship phase increased significantly if offered via digital technology. Considerably more participants (47% vs ∼20%) were interested in support from a peer cancer survivor /navigator if available through a digital app, in comparison to when the method was not specified. Similar findings were observed in the prior study with AYAs, where 82% expressed interest in support from a peer cancer survivor/navigator through a digital app, in comparison to 48.9% when the method was not specified.^32^ Participants were informed that could use to the digital app to connect and communicate with a peer cancer survivor/navigator, and that they would be able to view profiles of peers who matched their criteria and select the individual they felt was the best fit. These results suggest that technology-based support, and digital features such as profile viewing, choice in peer support provider, and digital communication, may significantly increase the appeal of peer support and peer navigation for some patients with HNC.

Governments have called for greater use of technology to extend the reach of cancer patient navigation.^27^ Technology may overcome many of the practical barriers to in-person support, as well as the communication and stigma-related barriers that patients with HNC face in particular.^21,40^ For example, technology could remove the need to travel to receive support (reducing logistical, mobility, and fatigue-related barriers), increase the convenience and timeliness of support (further reducing logistical barriers), and text-based communication could facilitate communication thereby reducing the stigma-and communication-related barriers associated with communication related impairments. Having a choice of support providers may also enhance patients’ perception of control and increase the likelihood of an appropriate match. These findings support previous research showing that patients prefer navigation delivered via home visits, telephone, and email to reduce stress and transportation barriers,^41^ and that having a choice of healthcare providers promotes patient autonomy and leads to support that is better aligned with patient preferences.^42^

Overall, preferences for communication with a peer cancer survivor/navigator were evenly divided between text-based and in-person formats. For example, similar proportions of participants reported wanting to communicate with a peer cancer survivor/navigator by email (47.4%) and in-person (46.6%). These findings are consistent with Caroll-Alfano who found that comparable proportions of laryngectomy patients participated in online support groups (52%) and in-person support groups (46%).^39^ In contrast, fewer participants in this study wanted to communicate by telephone (30%), and patients were least interested in communicating via social media or by video. Text versus audio communication may be preferred among some patients with HNC because of the impact of treatment on their ability to communicate verbally.

### 4.5. Interest in Being a Peer Navigator

A considerable proportion of participants (43%) expressed interest in being a volunteer peer navigator, and a similar proportion were willing to attend training for the role. This is an encouraging finding because peer navigation programs rely on the altruism of cancer survivors, and require clear role definition, appropriate training, and sustained support to be successful. It was notable, however, that 41% felt training was not needed, yet less than half who accessed peer support were satisfied with the peer support provided. These findings suggest that many patients may not understand the peer navigator role, and that the quality of existing peer support for patients with HNC varies. While navigator training varies in form and content,^43^ studies have shown that it is critical for the effectiveness of navigation services and more rigorous training leads to greater effect sizes.^24^ Encouragingly, participants who were interested in being peer navigators were 4.5 times more likely to rate training important.

### 4.6. Preferred Peer Matching Characteristics

According to optimal matching theory, the effects of social support will be greatest if matched to the demands of the stressor and the needs of the support seeker.^44^ However, administrators of HNC peer support programs in the United Kingdom have found optimal matching challenging given the limited pool but large diversity of the HNC population, along with the difficulty of remembering similar past patients.^22^ Digital apps can facilitate peer matching through matching algorithms based on participant preferences.^28^ In this study, the most important peer matching characteristics, endorsed by more than 50%, were related to clinical characteristics such as cancer type, treatments received, specific concerns, stage of disease and at age at diagnosis. Cancer type and specific concerns were also the topmost selected peer matching criteria in our study with AYAs with cancer,^32^ along with age at diagnosis. Matching based on sociodemographic characteristics (e.g. education, race/ethnicity, relationship status) was rated less important by most participants in both studies. However, in this study patients who identified as racialized were 2.5 times more likely to rate matching based on race/ethnicity important compared to patients who were white. This is an important finding, particularly given the higher rate of HNC in East and South Asian populations. Cancer patient navigation has been shown to be more effective in racialized populations when it includes culturally sensitive care.^45^

### 4.7. Predicting Need and Willingness to Provide Peer Navigation

Predicting who might need or want peer navigation could help timely referral referrals to navigation services and inform service planning and resource investments. In this study, participants who were older or who reported more anxiety were more likely to be interested in support from a peer navigator. In addition, we previously found that patients with HNC who were unpartnered, unemployed, and had more advanced cancer and/or more intense treatment, were more likely to suffer a “rocky treatment course” and would likely benefit from navigation.^46^ These findings are consistent with prior research among patients with cancer in the United States showing that sociodemographic factors (race, education, employment, income, insurance, and social support) as well as, physical and psychosocial functioning were associated with barriers to diagnostic resolution requiring navigation support.^47^ In contrast, participants who were interested in being peer navigators were those who felt as though they had adequate social support. These findings are consistent with prior research on prostate cancer peer navigators who reported high social support.^48^ By extension, prior research on cancer peer support providers suggests that they may also have higher physical functioning and emotional well-being compared to population norms.^49^ Collectively, these findings could inform the development of screening tools to inform timely referrals to peer navigation services and identify cancer survivors who may be interested in serving as peer navigators.

### 4.8. Limitations

The convenience sampling approach may have attracted participants that were more interested in peer support. However, a concerted effort was made to consecutively approach each eligible patient in clinic. In addition, offering patients the option to complete the survey on paper or online, may have extended the reach of the study, and included patients not comfortable using technology. Patient self-reported diagnosis and treatments often differed from the medical record. While diagnosis and treatment were verified for those recruited in person which constituted 93% of the sample; the accuracy of this data for the 12 recruited via email is unknown. The study was limited to patients who spoke English, and most patients who participated in the study identified as white (77%) and had completed college or university (72%); therefore, the findings may not reflect the perspectives of people who identify as racialized or who have lower levels of formal education. However, income level and comfort with and access to technology varied, suggestive of a somewhat socioeconomically diverse sample. Of note, while technology was not a barrier for most, 18% were not comfortable using technology and 12.5% did not have access to a computer or mobile device; therefore, a digital peer navigation program may not be suitable for all patients with HNC. Adaptations would be needed to ensure all patients who need or want peer navigation can access it. This would likely involve offering technology support and telephone-based access for individuals with limited comfort or access to computers. Lastly, further research is needed to explore the needs and preferences for peer support and peer navigation among a larger sample of people with lower socioeconomic status and in people of East and South Asian descent, given the higher incidence of HNC in these patient populations.

## 5. Conclusions

Among patients with HNC surveyed, half wanted to connect with other peers, about one-third did, and only one in five received peer support. Main barriers were privacy concerns associated with online support and logistical challenges associated with in-person programs. Technology-based communication with a peer cancer survivor or navigator was preferred by many and may overcome access barriers as well as offer peer matching based on patient preferences, however online privacy concerns must be addressed. A digital peer navigation program is a potentially feasible solution to improve access to peer support for patients with HNC, as well as support navigating the cancer journey. Given the limited research on peer support and navigation in patients with HNC relative to other cancer populations, these findings have the potential to fill a gap in the literature and guide the development of interventions tailored to their needs and preferences. Future research should explore the feasibility of technology-mediated peer navigation for patients with cancer, with adaptations to ensure that all patients who need or want peer navigation can access it.

## Data Availability

The datasets generated during and/or analyzed during the current study are available from the corresponding author on request.*

## Appendix

### Abbreviations

HNC: Head and neck cancer
HPV: Human papilloma virus
AYA: Adolescents and young adults
SPSS: Statistical package for the social sciences.
